# Cerebral Sulci as a Cause of Catheter Entry Point Deviation and Shunt Malposition and the Role of Surgical Navigation in Improving Accuracy

**DOI:** 10.7759/cureus.108515

**Published:** 2026-05-08

**Authors:** Naoki Wakuta, Yusuke Takemura, Ritsuro Inoue, Yoshinobu Horio, Kouhei Nii, Hiroshi Abe

**Affiliations:** 1 Department of Neurosurgery, Fukuoka University Chikushi Hospital, Fukuoka, JPN; 2 Department of Neurosurgery, Fukuoka Red Cross Hospital, Fukuoka, JPN; 3 Department of Neurosurgery, Fukuoka University Hospital, Fukuoka, JPN

**Keywords:** cerebral sulcus, neuronavigation, normal pressure hydrocephalus, shunt malposition, ventriculoperitoneal shunt

## Abstract

Background

In ventriculoperitoneal shunting for idiopathic normal pressure hydrocephalus, optimal trepanation sites and trajectories are critical to prevent shunt malposition. However, the effect of cerebral sulci at the burr hole site on unexpected intraoperative entry point changes remains unclear.

Methods

Preoperative computed tomography data from 50 patients with idiopathic normal pressure hydrocephalus (January 2018 to September 2024) were retrospectively reviewed. We analyzed the frequency of cerebral sulci beneath Kocher’s point necessitating entry point shifts and the distribution of alternative sites. Shunt malposition rates were compared between navigation-guided puncture on the basis of preoperative simulation and a conventional cohort using surface anatomical landmarks.

Results

Radiological evaluation of 100 bilateral Kocher’s points predicted minor puncture site adjustments in 20% of patients and major modifications, such as burr hole enlargement or relocation, were predicted in 17% of patients. In the conventional technique, the incidence of hemorrhage along the catheter tract was 7.4%, and the rate of suboptimal catheter tip placement was 33%. Introduction of a navigation system allowed preoperative identification of entry point shifts and trajectory planning. Consequently, no catheter tract hemorrhages occurred, and the rate of suboptimal placement was reduced to 13.0%.

Conclusion

Unanticipated cerebral sulci at conventional entry points are negative confounding factors for successful ventricular puncture. Preoperative identification of appropriate entry points and trajectories is important for surgical success. Recognizing such anatomical variations through preoperative review of standard radiological images is essential for improving accuracy and safety, even when technological aids are limited. Furthermore, the implementation of a navigation system provides critical support in achieving these objectives and reducing malposition rates.

## Introduction

Ventriculoperitoneal (VP) shunt surgery is the standard treatment for idiopathic normal pressure hydrocephalus (iNPH). Ventricular catheter misplacement has been reported as a major factor associated with shunt malfunction [1,2]. Therefore, frontal ventriculostomy is an important surgical process consisting of cannulation of the lateral ventricles. Kocher’s point (KP) is a common landmark named after the neurosurgeon Emil Theodor Kocher and is used to determine one of the major ideal entry points for ventriculostomy in the frontal horn of the lateral ventricle [3,4]. Although multiple studies have been conducted to improve the accuracy of ventriculostomy, most focused on determining the theoretical optimal puncture trajectory [5-9]. However, the local sulcal anatomy at the ventriculostomy’s entry point has rarely been examined and discussed before surgery. An unanticipated change in the entry point to avoid this anatomical feature may lead to an inappropriate trajectory and poor shunt placement. Therefore, we investigated the incidence of cerebral sulci beneath KP using radiological data from clinical cases of iNPH and examined how the implementation of surgical navigation could improve puncture accuracy by addressing these anatomical factors.

## Materials and methods

Study design

Consecutive adult patients in whom primary VP shunt surgery via frontal horn puncture was performed for iNPH at Fukuoka Red Cross Hospital from January 2018 to September 2024 were included. This study was approved by the institutional review board and regional ethics committee of the Fukuoka Red Cross Hospital (Approval code: 24021), and was conducted in accordance with the Declaration of Helsinki of 1975, as revised in 2000. Patients with a history of direct neurosurgery or dementia, hydrocephalus due to other brain diseases, or cranial abnormalities such as malformations were excluded. All included patients were diagnosed by typical symptomatic ventricular enlargement with an Evans index >0.3, and the cerebrospinal fluid tap-test was performed to predict the clinical response for VP shunt placement. We retrospectively evaluated preoperative plain head computed tomography data through the study period to assess anatomical features using the Brainlab Curve2 neuronavigation system (Brainlab AG, Munich, Germany) and its Brainlab Elements software. However, during the study period, shunt surgery was performed by referring to classical surface anatomical landmarks (the nasion, inion, ipsilateral medial canthus, and ipsilateral external auditory meatus) only until May 2022 (group C), and we then used intraoperative navigation (group N) from June 2022 onwards with introduction of the neuronavigation system.

Radiological study

**Figure 1:**
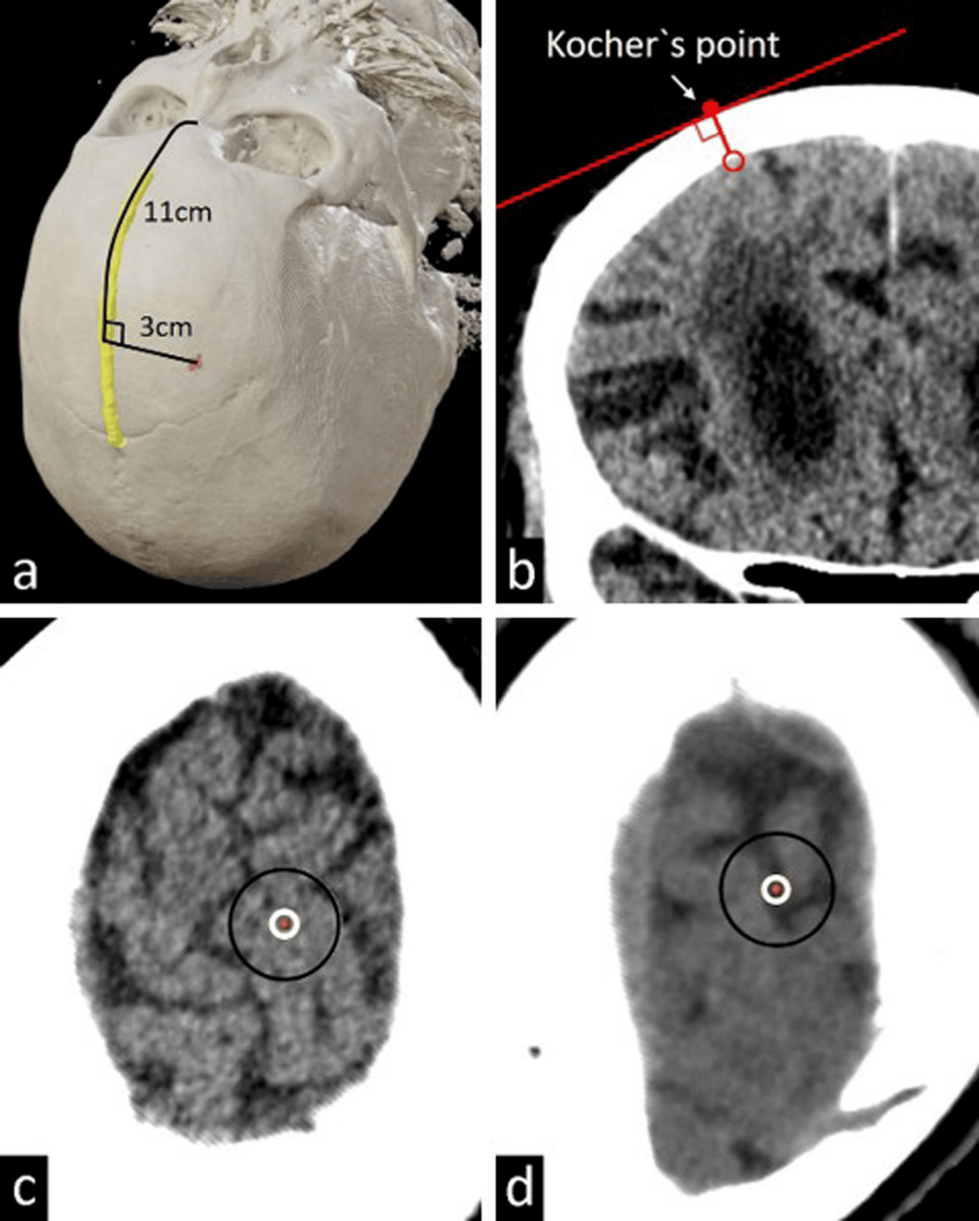
Computed tomography images with Brainlab Elements software. Kocher’s point was determined as 11 cm posteriorly from the nasion along the skull surface and 3 cm laterally from the midline (a). The reference entry point was detected as the intersection of a perpendicular line from Kocher’s point and the cerebral surface (b). A case of a sufficient margin to reference the entry point from the cerebral sulci (c). A case that required adjustment of the entry point for ideal ventriculostomy (d). White circle: 2.5-mm radius from the reference entry point. Black circle: 7.5-mm radius from the reference entry point (burr hole edge).

In this study, KP was determined to be 11 cm superior and posterior from the nasion and 3 cm lateral from the midline, as described previously [3,4,10]. The sagittal suture and its extensions as the midline, coronal suture, and nasion were identified to determine the locations of bilateral KPs using Brainlab Elements software (Figure 1). The baseline reference entry point (REP) to puncture the cortex for ventriculostomy was defined as the intersection of a perpendicular line from KP and the cerebral surface. The ideal trajectory of ventriculostomy was defined as the line from the entry point to the ipsilateral foramen of Monro via the frontal horn. If there were cerebral sulci, which are an inappropriate condition for ideal ventriculostomy, running nearby the REP or intersecting on the trajectory line, the alternative entry point (AEP) for suitable puncture was searched for concentrically around the REP. The distance from the REP to the AEP was then measured. Taking into consideration the 2.7-mm ventricular catheter diameter and safety margin for the surgical procedure, we defined the AEP as follows: 1) a cortex greater than 2.5 mm away from the edge of cerebral sulci, 2) the closest point from the REP, and 3) where the ideal trajectory for ventriculostomy can be maintained. The diameter of the burr hole made by the perforator in our hospital is 15 mm (radius: 7.5 mm). Therefore, an AEP less than 5.0 mm away from the REP required a minor adjustment in the burr hole at KP. However, more than 5.0 mm from the REP indicated that major adjustment with burr hole enlargement or another burr hole opening would be required.

Surgical techniques

**Figure 2:**
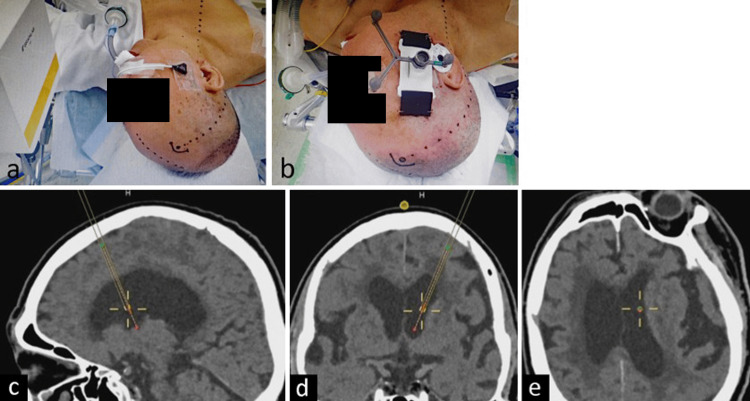
Surgical navigation setups and ventriculostomy following the planned trajectory. Surgical setups with electromagnetic navigation (a) or optical navigation with a stick-on reference frame using a fixation device (b). Ventriculostomy was performed by tracing the trajectory planned preoperatively (sagittal view (c), coronal view (d), catheter eye view (e)).

To perform the VP shunt, the right hemisphere generally corresponding to the non-dominant side was mainly selected, and a decision was made at our neurosurgery meeting. All patients underwent a VP shunt in accordance with the standard procedure in Japan, and a Codman CERTAS Plus adjustable shunt valve with SiphonGuard (Integra Life Sciences, Princeton, NJ, USA) was implanted. Informed consent was obtained from all patients before surgery. In group C, a burr hole opening was performed at the manually measured KP by referring to anatomical surface landmarks, and ventriculostomy was performed according to the ipsilateral medial canthus and external auditory meatus. The shunt insertion length was 5.5 to 6.5 cm, which was decided at the discretion of the surgeon. In group N, the burr hole location was determined on the basis of preoperative planning with Brainlab software. Either magnetic or optical surgical navigation was selected randomly depending on which was available for each procedure, and both were capable of required head position changes during surgery with the use of a stick-on reference array (Figure 2). Ventriculostomy was performed by tracing the planned trajectory. The ventricular catheter tip position was evaluated by referring to a previously described protocol: 1) grade 1, floating in cerebrospinal fluid without contact with the ventricular walls; 2) grade 2, touching the ventricular wall; and 3) grade 3, within the parenchyma or complete misplacement in which shunt revision surgery was required [11]. Complications were defined as any deviation from the normal postoperative course within 30 days of surgery, and shunt tip position grade 3 was equivalent to shunt malfunction during the study period.

Endpoint evaluation

The primary endpoint was to assess the potential risk of requiring adjustment of the entry point from the REP to avoid cerebral sulci. The secondary endpoint was to evaluate the shunt malposition risk associated with entry point adjustments, with and without the use of intraoperative navigation.

Statistical analysis

Statistical analysis was performed using R software (version 4.5.2; R Foundation for Statistical Computing, Vienna, Austria). Continuous variables are presented as the mean ± standard deviation. Comparisons between groups were performed using Fisher’s exact test for categorical values and the Mann-Whitney U test for continuous values. A p value < 0.05 was considered statistically significant.

## Results

**Figure 3:**
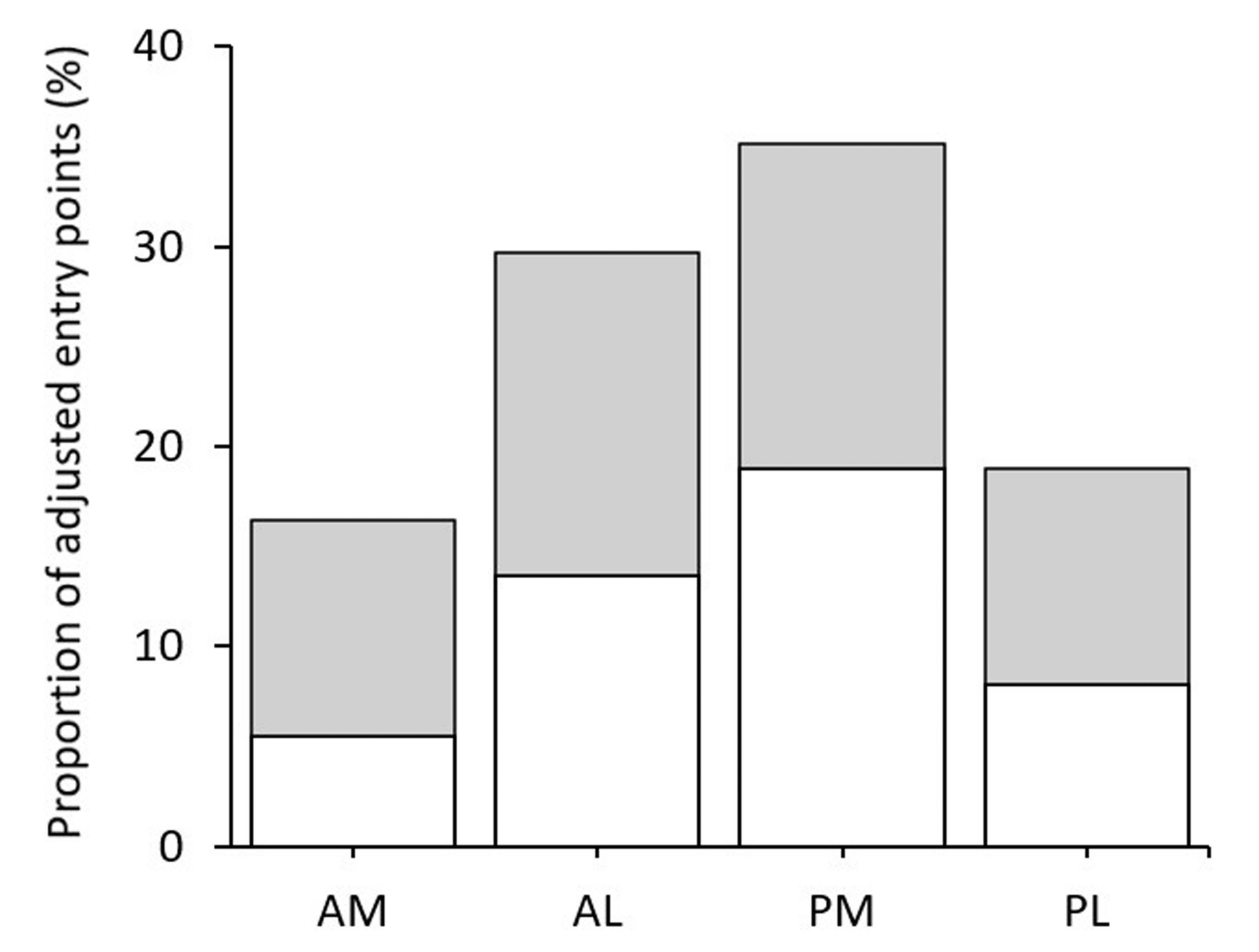
Proportion of the alternative entry point around the reference entry point. White bars: percentage on the right side; gray bars: percentage on the left side. AM: anteromedial region, AL: anterolateral region, PM: posteromedial region, PL: posterolateral region.

Fifty patients were included. The mean age was 73.0 ± 10.9 years old and 28 (56.0%) were men. The patients’ characteristics were generally similar between the two groups, with no significant differences between them, except for the prevalence of diabetes mellitus (Table 1). The left hemisphere was selected in three patients because their ventral tube ran on the left side owing to a history of thoracic or abdominal surgery. An adjustment from the REP for appropriate ventriculostomy was suggested in 37 sides of 33 patients, especially major adjustment in 17 sides of 16 patients (Table 2). The frequency of adjusting the entry point showed no significant laterality; in 4% of patients, no ideal trajectory was achievable regardless of the REP side selected. The distribution of the AEP ranged between a radius of 2.5 and 9.4 mm (mean: 4.5 ± 2.2 mm) away from the REP, and it appeared to be further on the left side (right vs left: 4.4 ± 2.2 vs 6.2 ± 2.2 mm), but this was not significant (p = 0.648). Thirteen (35.2%) AEPs were on the posteromedial side and 11 (29.7%) AEPs were on the anterolateral side (Figure 3).

**Table 1:** Patients’ baseline characteristics and clinical outcomes of ventriculoperitoneal shunt surgery. Data are shown as the mean ± SD or n (%). Comparisons between groups were performed using Fisher’s exact test for categorical values and the Mann–Whitney U test for continuous values. For the Mann–Whitney U test, test statistics were U = 261 (age) and U = 202.5 (operative time). A p value < 0.05 was considered statistically significant. Abbreviations: SD: standard deviation.

Variable	Group C	Group N	p value
Sex, Male/female	15 (55.6)/12 (44.4)	13 (56.6)/10 (43.4)	1.000
Age, years	72.0 ± 11.7	74.0 ± 9.7	0.324
Comorbidity			
Hypertension	13 (48.2)	12 (52.3)	1.000
Diabetes mellitus	2 (7.4)	10 (43.4)	0.006
Dyslipidemia	3 (11.1)	5 (21.7)	0.444
Surgical procedures			
Right side/left side	25 (92.6)/2 (7.4)	22 (95.7)/1 (4.3)	1.000
Operative time (minutes)	72.0 ± 16.6	85.0 ± 18.2	0.026
Ventricular catheter position			0.528
Grade 1	18 (66.7)	20 (87.0)	
Grade 2	7 (25.9)	2 (8.7)	
Grade 3	2 (7.4)	1 (4.3)	
Complication			
Ventriculostomy-associated hemorrhage	2 (7.4)	0 (0)	0.493
Overshunting with subdural hematoma	1 (3.7)	2 (8.7)	0.588
Shunt infection	1 (3.7)	0 (0)	1.000
Revision surgery	3 (11.1)	1 (4.3)	0.614

**Table 2:** Comparison of the puncture entry point on the cerebral cortex for ideal ventriculostomy. Data are shown as n (%). Abbreviations: AEP: alternative entry point, REP: reference entry point.

Variable	Right side	Left side	Total
REP	33 (66)	30 (60)	63 (63)
AEP ≤ 5.0 mm from the REP	11 (22)	9 (18)	20 (20)
AEP > 5.0 mm from the REP	6 (12)	11 (22)	17 (17)

Evaluation of operative data in group C showed that the retrospectively recommended entry point in the operated site was the REP in 17 (63.0%) patients and the AEP in 10 (37.0%) patients. However, 14 patients whose operated site was the REP actually underwent puncture at the REP. Of these, two resulted in grade 2 catheter tip positioning. Additionally, the burr hole was technically misplaced from KP in the remaining three patients, which led to one grade 2 and one grade 3 catheter tip positioning that required shunt revision surgery. In the remaining 10 patients, the burr hole was located at KP, and minor adjustments were required in six (22.2%) and major adjustments in four (14.8%) patients. These proportions were consistent with the results of the radiological assessment (Table 2). Of these, grade 2 positioning occurred in two of the six patients who required minor adjustment and in two of the four patients who required major adjustment. Grade 3 positioning occurred in one of the patients who required major adjustment. In patients whose optimal entry point was the REP, the rate of grade 2 or 3 shunt tip positioning was lower than that in those with an unanticipated change in the entry point (14.3% vs 53.9%). Regarding the ventricular catheter position in group C, grades 1, 2, and 3 accounted for 66.7%, 25.9%, and 7.4% of patients, respectively.

In group N, shunt malposition of grade 3 occurred in one patient in whom intraoperative navigation malfunctioned caused by misattachment of the reference frame. Trepanation and ventriculostomy were performed as simulated preoperatively in other cases, and there were no cases of shunt malposition, but two cases were grade 2 because of a slight curve of the catheter tip. There were no complications associated with introduction of the navigation. The mean surgical time was 72 ± 16.6 minutes in group C and 85 ± 18.2 minutes in group N. The extended time was 13 minutes.

## Discussion

Most previous studies on improving misplacement of a ventricular catheter focused on modification of the classical puncture point or on new anatomical landmarks for the ideal puncture trajectory and discussed the angle and/or length of the course [5-9]. Unanticipated cerebral sulci at these conventional entry points can act as negative confounding factors that hinder the reproduction of such ideal surgical outcomes by necessitating unplanned intraoperative adjustments that deviate from the intended trajectory. However, the clinical significance of these anatomical variations has rarely been investigated. Robertson et al. evaluated the risk of hemorrhage in ventriculostomy with radiographic information of cerebral veins in 50 patients, but their data were based on normal ventricular anatomy [12]. Cerebral sulci are often enlarged in older patients because of cerebral atrophy, whereas they may present as tightened sulci at high convexity in iNPH cases. Therefore, practical assessment using non-disease-specific radiological images or cadavers is insufficient. Only the analysis of actual clinical data from iNPH can result in surgically relevant and applicable findings. To the best of our knowledge, this is the first study to investigate cerebral sulci, which affect the entry point for ventriculostomy at KP, based on radiographical data of clinical iNPH cases.

We found that the ideal trajectory could be visualized from typical puncture points at KP in more than 60% of the patients. However, 17% of the patients required burr hole enlargement or another burr hole opening. This study suggested that the posteromedial region of the REP could be used to detect the AEP regardless of the side. However, surgeons should recognize that relocating the entry point may bring the surgical site closer to the superior sagittal sinus medially and the primary motor cortex posteriorly.

Previous studies on shunt malplacement have reported that changing the burr hole position is one of the factors related to the failure of ventriculostomy [10,13]. In our study, the distribution of catheter positions in group C (grades 1, 2, and 3: 66.7%, 25.9%, and 7.4%, respectively) aligns closely with previous findings [13]. Notably, the incidence of grades 2 and 3 was higher in group C than in group N (Table 1). Our clinical data in the two groups also indicated that relying solely on intraoperative classical anatomical landmarks may lead to inaccurate identification of the optimal entry point and/or an inaccurate trajectory direction during surgery. This inaccuracy could reduce the theoretical success rate of appropriate ventriculostomy. Regarding the puncture direction, cannulating the ventricle at a 90° angle to the skull is a method used for successful ventriculostomy [14]. However, the acceptable range of entry points for ensuring an ideal trajectory using this method has been reported to be limited [15]. Therefore, this method is not always reliable. Rehman et al. reported that 10.4% of perpendicular catheter insertions were placed in the brain parenchyma even from KP [8]. Other recent studies also showed that the correction angle was often required to perform ideal ventriculostomy [7,16]. However, no previous studies have established landmarks that ensure flexible and accurate ventricular access when the entry site is adjusted.

Neurosurgeons often accept that there will be some uncertainty in the entry point and trajectory, and consider the risk of misplacement as an unavoidable part of the procedure as long as the probability of such complications remains sufficiently low [17]. This risk can be addressed with the introduction of navigation. In this study, with the exception of one case of a technical mistake in the navigation settings, no shunt malposition requiring revision was observed in any patients who were operated as preoperatively simulated in group N. Previous studies have reported that the introduction of navigation systems considerably reduces shunt misplacement, failure, and revision rates [2,11,18]. A reduction in shunt revisions provides a clear clinical benefit by protecting patients from the risks associated with additional surgical invasiveness and potential complications. Furthermore, the implementation of navigation does not increase the surgical burden on the patient. In conventional ventriculoperitoneal shunt procedures, surgeons may occasionally lose an accurate trajectory for ventriculostomy when relying solely on surface landmarks, often because of head rotation. However, with the aid of navigation, an appropriate puncture can be performed by tracing the ideal trajectory in real time while monitoring the display.

There are also several issues of the use of surgical navigation in routine shunt surgery, such as the need for head fixation, an increase in the procedural time, and additional cost implications. Rigid head fixation in optical navigation increases the complexity of setup and limits head mobility in the operation. Therefore, using a stick-on reference array or magnetic navigation is recommended, as used in the present study (Figure 2). Prolongation of the operation time is another issue at the application of navigation. A survey on ventriculostomy practices reported that 94% of neurosurgeons were reluctant to use technological aids if they added more than 10 minutes to the procedure [17], and Hayhurst et al. reported an acceptable additional five to 10 minutes for the use of navigation [11]. In the present study, the mean surgery time for group N showed a significant extension of 13 minutes compared with group C. However, this duration is comparable to the result of another study using the BrainLab navigation system [15]. The cost-benefit ratio with installation of the navigation system must be carefully assessed. However, elimination of avoidable complications and shunt revisions is beneficial in cost saving and for the patients. For institutions where navigation systems are not readily available, a meticulous preoperative review of standard radiological images, such as plain computed tomography or magnetic resonance imaging, to identify the spatial relationship between the intended entry point and the underlying cerebral sulci, is a practical alternative. Identifying the need for entry point shifts through such simulations may help reduce the risk of unanticipated complications. Performing further research to define more accurate anatomical landmarks that ensure ideal ventriculostomy, even from the AEP, may provide another solution.

There are some limitations to our study. First, the retrospective nature of this study was prone to selection bias. Second, our preoperative radiological assessments relied solely on routine non-enhanced computed tomography to accommodate the reduced renal function frequently observed in older patients with iNPH. Based on the fact that cerebral sulci often correspond to the course of vessels, the lack of contrast-enhanced imaging restricted our ability to perform a detailed evaluation of the vascular anatomy. Consequently, while our findings may be relevant to the risk of vascular injury, the scope of this assessment remains limited. Finally, our clinical outcomes may have been subject to bias because of the small sample size, but it is comparable to those of previous reports [13,19].

## Conclusions

In this study, we evaluated the cerebral sulci as an anatomical risk leading to catheter entry point deviation and shunt malposition, and showed that this risk could be mitigated through certain systematic adjustments. Furthermore, we showed that preoperative planning with a navigation system facilitates the optimization of puncture sites and trajectories, leading to improved clinical outcomes. Although issues remain in the implementation of this navigation, further research is warranted to identify more flexible and dependable anatomical landmarks for achieving ideal ventriculostomy from adjusted entry points.

## References

[1] Feletti A, d'Avella D, Wikkelsø C (2019). Ventriculoperitoneal shunt complications in the European idiopathic normal pressure hydrocephalus multicenter study. Oper Neurosurg.

[2] Golubovsky JL, Liao J, Hogue O (2022). Complications associated with ventriculoperitoneal shunt surgery for normal pressure hydrocephalus using stereotactic navigation and abdominal laparoscopy: a single-institution case series. Oper Neurosurg.

[3] Hildebrandt G, Surbeck W, Stienen MN (2012). Emil Theodor Kocher: the first Swiss neurosurgeon. Acta Neurochir (Wien).

[4] Morone PJ, Dewan MC, Zuckerman SL, Tubbs RS, Singer RJ (2020). Craniometrics and ventricular access: a review of Kocher's, Kaufman's, Paine's, Menovksy's, Tubbs', Keen's, Frazier's, Dandy's, and Sanchez's points. Oper Neurosurg.

[5] Kassam AB, Monroy-Sosa A, Fukui MB (2020). White matter governed superior frontal sulcus surgical paradigm: a radioanatomic microsurgical study—part II. Oper Neurosurg.

[6] Park B, Han S, Byoun HS, Han S, Choi SW, Lim J (2020). The assessment of geometric reliability of conventional trajectory of ventriculostomy in a three dimensional virtual model and proposal of a new trajectory. Neurol Med Chir (Tokyo).

[7] Park J, Son W, Park KS, Kim MY, Lee J (2016). Calvarial slope affecting accuracy of Ghajar Guide technique for ventricular catheter placement. J Neurosurg.

[8] Rehman T, Rehman Au, Ali R (2013). A radiographic analysis of ventricular trajectories. World Neurosurg.

[9] Vigo V, Tassinari A, Scerrati A, Cavallo MA, Rodriguez-Rubio R, Fernandez-Miranda JC, De Bonis P (2022). Ideal trajectory for frontal ventriculostomy: radiological study and anatomical study. Clin Neurol Neurosurg.

[10] Woo PY, Wong DK, Yuan Y (2022). A morphometric analysis of commonly used craniometric approaches for freehand ventriculoperitoneal shunting. Oper Neurosurg.

[11] Hayhurst C, Beems T, Jenkinson MD (2010). Effect of electromagnetic-navigated shunt placement on failure rates: a prospective multicenter study. J Neurosurg.

[12] Robertson FC, Abd-El-Barr MM, Mukundan S Jr, Gormley WB (2017). Ventriculostomy-associated hemorrhage: a risk assessment by radiographic simulation. J Neurosurg.

[13] Wan KR, Toy JA, Wolfe R, Danks A (2011). Factors affecting the accuracy of ventricular catheter placement. J Clin Neurosci.

[14] O'Leary ST, Kole MK, Hoover DA, Hysell SE, Thomas A, Shaffrey CI (2000). Efficacy of the Ghajar Guide revisited: a prospective study. J Neurosurg.

[15] Yamada SM, Yamada S, Goto Y, Nakaguchi H, Murakami M, Hoya K, Matsuno A (2012). A simple and consistent technique for ventricular catheter insertion using a tripod. Clin Neurol Neurosurg.

[16] Beckett JS, Gaonkar B, Babayan D (2018). Autonomous trajectory planning for external ventricular drain placement. Oper Neurosurg.

[17] Amoo M, Henry J, Javadpour M (2021). Common trajectories freehand frontal ventriculostomy: a systematic review. World Neurosurg.

[18] Jung N, Kim D (2013). Effect of electromagnetic navigated ventriculoperitoneal shunt placement on failure rates. J Korean Neurosurg Soc.

[19] Stieglitz LH, Giordano M, Samii M, Luedemann WO (2010). A new tool for frameless stereotactic placement of ventricular catheters. Neurosurgery.

